# Integrative analyses of genes related to liver ischemia reperfusion injury

**DOI:** 10.1186/s41065-022-00255-8

**Published:** 2022-10-18

**Authors:** Hang-Pin Wang, Chu-Hong Chen, Ben-Kai Wei, Ying-Lei Miao, Han-Fei Huang, Zhong Zeng

**Affiliations:** 1 Organ Transplantation Center, the First Affiliated Hospital of Kunming Medical University, Kunming, 650032 China; 2 Department of Gastroenterology, the First Affiliated Hospital of Kunming Medical University, Kunming, 650032 China; 3 Yunnan Province Clinical Research Center for Digestive Diseases, Kunming, 650032 China

**Keywords:** Liver ischemia reperfusion injury (LIRI), Differentially expressed gene (DEG), Network analysis, Protein-protein interaction

## Abstract

**Background:**

Liver ischemia reperfusion injury (LIRI) is not only a common injury during liver transplantation and major hepatic surgery, but also one of the primary factors that affect the outcome of postoperative diseases. However, there are still no reliable ways to tackle the problem. Our study aimed to find some characteristic genes associated with immune infiltration that affect LIRI, which can provide some insights for future research in the future. Therefore, it is essential for the treatment of LIRI, the elucidation of the mechanisms of LIRI, and exploring the potential biomarkers. Efficient microarray and bioinformatics analyses can promote the understanding of the molecular mechanisms of disease occurrence and development.

**Method:**

Data from GSE151648 were downloaded from GEO data sets, and we performed a comprehensive analysis of the differential expression, biological functions and interactions of LIRI-associated genes. Then we performed Gene ontology (GO) analysis and Kyotoencydlopedia of genes and genomes (KEGG) enrichment analysis of DEGs. At last, we performed a protein-protein interaction network to screen out hub genes.

**Results:**

A total of 161 differentially expressed genes (DEGs) were identified. GO analysis results revealed that the changes in the modules were mostly enriched in the neutrophil degranulation, neutrophil activation involved in immune response, and neutrophil mediated immunity. KEGG enrichment analysis of DEGs demonstrated that LIRI mainly involved the cytokine-cytokine receptor interaction. Our data indicated that macrophages and neutrophils are closely related to LIRI. 9 hub genes were screened out in the protein-protein interaction network.

**Conclusions:**

In summary, our data indicated that neutrophil degranulation, neutrophil activation involved in immune response, neutrophil mediated immunity and cytokine-cytokine receptor interaction may play a key role in LIRI, HRH1, LRP2, P2RY6, PKD1L1, SLC8A3 and TNFRSF8, which were identified as potential biomarkers in the occurrence and development of LIRI. However, further studies are needed to validate these findings and explore the molecular mechanism of these biomarkers in LIRI.

## Introduction

Currently, the most suitable treatment for most end-stage liver diseases is liver transplantation, and the success rate of liver transplantation has increased greatly thanks to advances in aseptic and surgical methods and immunosuppressive techniques [1]. However, some liver transplantation operations are unsuccessful, and the survival time after liver transplantation is still short, which is closely related to the liver ischemia-reperfusion injury (LIRI) during the operation. LIRI refers to the aggravation of cellular structure and function damage, and further deterioration of organ function after liver tissue ischemia and reperfusion [2]. LIRI includes ischemia liver injury and subsequent reperfusion injury. It is not only the pathophysiological process of hepatectomy and liver transplantation, but also the inevitable result of organ recovery, cold ischemia time, and reperfusion after transplantation. The major cause is the initial injury of the donors’ organs and brain death, followed by allograft loss of vascularization at the time of procurement, and then the reperfusion of the recipient organ leading to further injury [3]. In addition, LIRI is a sterile inflammatory response driven by innate immunity [4]. IRI can activate IL1 receptor-associated kinase 4 (IRAK-4), leading to activation of transcription factors, such as NF-κB, and the upregulation of tumor necrosis factor-α (TNF-α), both of which lead to inflammation. In addition, transforming growth factor-β-activated kinase (TAK) may also enhance liver inflammation by activating the NF-κB pathway. Thus, Thus, dual-specificity phosphatase (DUSP) [5], an inhibitor of TAK1, was found to be effective in alleviating IRI-induced inflammation in mice. As reported, lipid metabolism disturbance induces inflammation, oxidative stress, apoptosis, and autophagy. in an understandable against hepatic ischaemia-reperfusion injury via Tak1 suppression. Arachidonate 12-lipoxygenase (ALOX12) was found to be up-regulated in the liver after IRI and could induce inflammation by activating MAPK and NF-κB pathways [6].

Metabolic disorders, large amounts of reactive oxygen species (ROS), and cytokines or chemokines can stimulate severe inflammatory responses of immune cells, which promotes the full development of inflammatory hepatocellular injury. Damage-associated molecular patterns (DAMPs) can stimulate myeloid and dendritic cells via pattern recognition receptors (PRRs) to initiate the immune response [7]. Acidic microenvironment plays an important role in the progression of LIRI. Due to the accumulation of acidic substances such as ketone bodies and lactic acid, the acidic microenvironment promotes hepatocyte injury associated with IRI. High levels of lactate act as Damage-Associated Molecular Patterns (DAMPs), thereby promoting inflammation during the reperfusion phase. In addition, intracellular acidosis causes an imbalance in protein turnover, leading to enzyme inhibition and destruction of important proteins, and prevents ATP reserve reorganization after reperfusion. Acidic microenvironment not only inhibits the generation and function of CD4 + CD25 + Foxp3+ iTregs through PI3K/AKT/mTOR signaling [8], it also triggers upregulation of NO synthetase in macrophages, accumulation of neutrophils, inactivation of cytoplasmic and membrane-related enzymes, and down-regulation of cAMP, protein, and DNA synthesis [9]. LIRI is a key factor associated with liver transplantation, liver resection, trauma, and hemorrhagic shock [10]. Previous studies have shown that LIRI is involved in a variety of mechanisms, including the complement activation, neutrophils and Kupffer cells activation, Ca^2+^ overload, pH imbalance, endothelin (ET)/ nitric oxide (NO) ratio imbalance, mitochondrial damage caused by oxygen free radicals, liver microcirculation dysfunction, and the influence of various cytokines [11]. HSCs are activated and proliferated by IRI, possibly through signals from Kupffer cells. HSCs promote early LIRI by limiting ROCK-mediated hepatic microcirculation, the effect of ET-1 signaling, and the pro-inflammatory cascades triggered by TNF-α. In the repair phase of LIRI, HSCs also regulate fibrogenesis, and its extent may be crucial for the functional recovery of the liver [12]. Matrix Metalloproteinases (MMPs), Neutrophil Gelatinase-associated Lipocalin (NGAL) and Mitochondrial flavin mononucleotide (FMN) might become reliable biomarkers of LIRI [13]. The mechanism of LIRI is complex, and involves multiple lncRNAs and miRNAs, they both regulate the expression of mRNAs through various mechanisms. Numerous miRNAs have been confirmed to be associated with apoptosis, autophagy, oxidative stress and cellular inflammation that accompany HIRI pathogenesis,such as AK139328, CCAT1, MALAT1, TUG1 and NEAT1 [14]. Although many key factors in the pathogenesis of LIRI have been identified, the complete mechanism and treatment of hepatic IRI have not been fully understood. Recent studies have explored that the activation of immune cells to induce inflammation is crucial in the occurrence and development of LIRI. Liver resident immune cells such as Kupffer cells and dendritic cells release cytokines and chemokines in response to the stimulation of injury-related molecular patterns such as HMGB1 or DNA/RNA, which activate neutrophils, T monocytes and lymphocytes, to form inflammatory responses and aggravation of tissue damage [15]. Of these, liver interstitial dendritic cells are heterogeneous innate immune cells, which is important to the induction, integration and regulation of inflammation after liver transplantation. Interstitial dendritic cell also participates in the regulation of IRI and anti-donor immunity. However, there is no therapeutic options are available to mitigate IRI [16]. Therefore, to tackle this situation, new genes and immune cells for LIRI are required to investigate. We aimed to identify characteristic genes associated with immune infiltration that affect LIRI, search the therapeutic targets for immune cells to treat the liver ischemia-reperfusion injury through the cells, molecules and involved pathways regulated by these genes, and alleviate the severity of liver ischemia-reperfusion injury, to improve the success rate and late survival rate of liver transplantation, and provide some insights for the future research of liver diseases.

## Materials and methods

### Microarray data

GEO (http://www.ncbi.nlm.nih.gov/geo/) is a public database with high-throughput gene expression data, chips and microways [17]. We downloaded the sample information from the GEO to identify the candidate genes in LIRI. GSE151648 database (https://www.ncbi.nlm.gov/geo/query/acc.cgi?acc=GSE151648) was selected for the study based on the key word “Liver ischemia reperfusion injury”. For the Expression Matrix in the GSE151648, Ensembl gene IDs were converted to Hgnc symbols using BiomaRt (http://www.biomart.org) package in R [18]. The data type was expression profiling by high throughput sequencing, and the organism was *Homo sapiens*. The GSE151648 dataset contained the RNA-seq data of 23 IRI^+^ patients and 17 IRI^−^ patients before and after transplantation respectively.

### Identification and analyses of DEGs

Kyoto encyclopedia of genes and genomes (KEGG) is a database resource for understanding high-level functions and biological systems from large-scale molecular datasets generated by high-throughput experimental technologies [19]. Gene Ontology (GO) analysis including biological process (BP), molecular functions (MF), and cellular components (CC), was used to annotate genes and analyze the biological process of genes. Differential expression analyses of IRI^+^ and IRI^−^ groups were performed using the edgeR R package. Expression profiles of IRI^+^ patients and IRI^−^ patients were compared to identify the DRGs. Moreover, genes were obtained from each sample based on the following criteria: |log2 (fold-change)| > 1, *P* value< 0.05. The screened Differentially Expressed Genes (DEGs) were used to create a volcano map and the top 20 up-regulated and down-regulated DEGs were used to plot a heat map by pheatmap.1 package. The expression matrices of these two groups were transformed into TPM-normalized expression matrix.

### LASSO regression analysis and SVM-RFE analysis

In this study, the least absolute shrinkage and selection operator (LASSO) logistic regression conducted by R package glmnet and SVM-RFE (SVM-Recursive Feature Elimination) analysis conducted by SVM-RFE algorithm were applied to the differential gene expression matrix obtained in the previous step. Then a Venn diagram was drawn to show the intersection of the genes obtained in the previous step. VennDiagram package was used to generate high-resolution Venn and Euler plots [20]. The ROC curves were plotted based on the intersection genes in the Venn diagram to evaluate the efficiency of the intersection genes in differentiating IRI^+^ and IRI^−^ samples. The boxplot was drawn according to the expression levels of the intersection genes and Wilcoxon test (non-parameter), namely wilcox.test function, was used to compare the differences of genes in the two groups.

### Differential analysis of immune cell infiltration types

The ssGSEA analysis of R package gsva [21] was performed between the IRI^+^ and the IRI^−^ groups to analyze the significant differential expression of them. To further investigate the relationship between different types of immune cell infiltration and the expression of characteristic genes, Pearson correlation coefficient was applied. These genes involved in immune cell infiltration were the potential immune cell therapeutic targets for the treatment of liver ischemia reperfusion injury.

### Correlation analysis of characteristic genes and ssGSEA scores

To identify the relevant immune cell regulation situations and impacts of genes in IRI, we performed Spearman correlation analysis on the expression level (TPM) of the 14 characteristic genes and ssGSEA scores of IRI+ and IRI- samples. The lollipop map of the correlation between the 14 characteristic genes and 24 sorts of immune cells was obtained.

### PPI network construction and module analyses

The interactions of 14 DEGs were determined using the STRING online database (https://string-db.org/) with a combined score > 0.4 to identify physical PPIs among nodes underlying IRI [22]. The obtained data were then evaluated using Cytoscape software [23].

## Results

### LIRI associated DEGs

**Fig 1:**
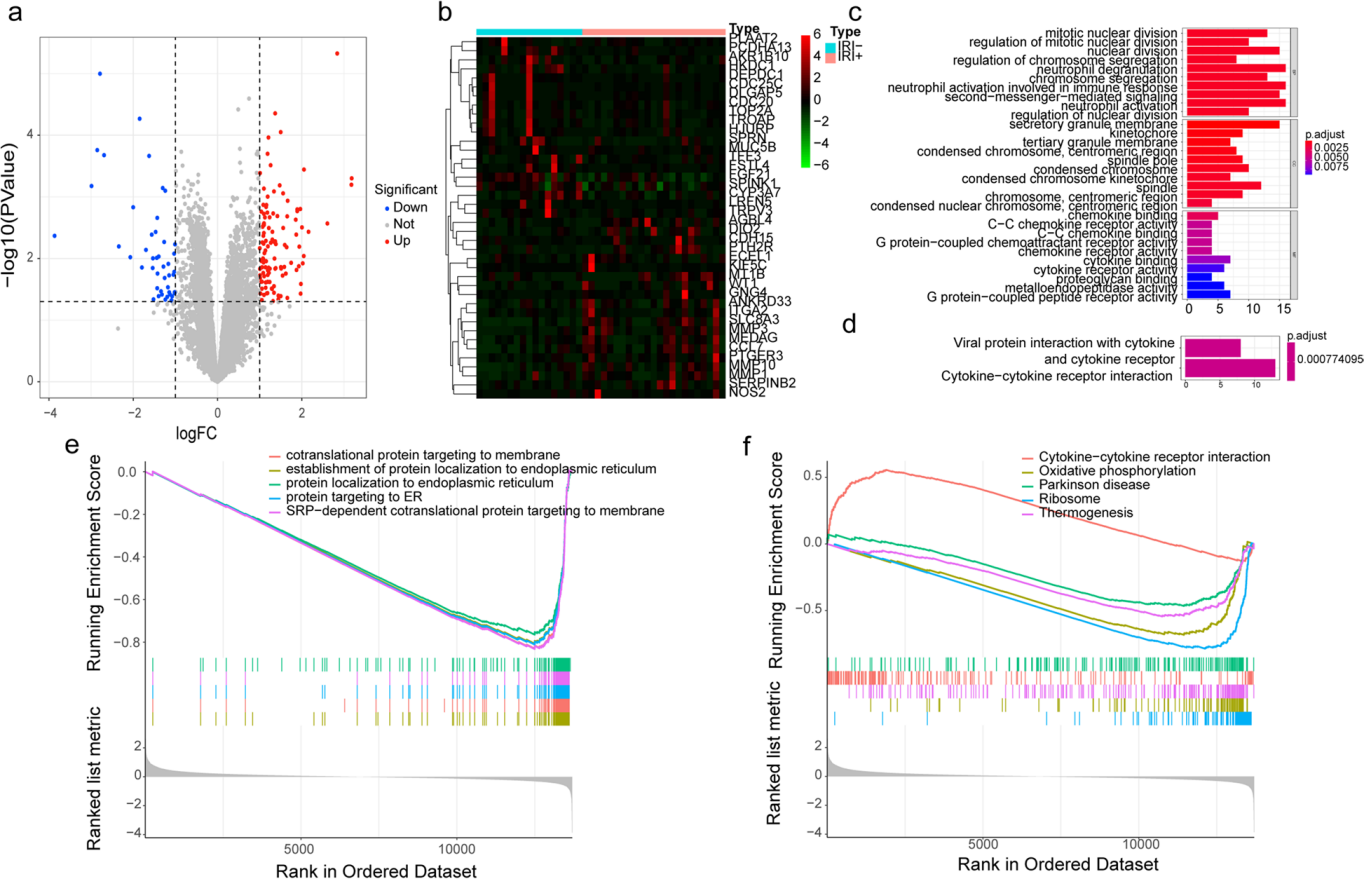
Analyses of DEGs of IRI+ and IRI- groups. **a** Volcano plot of the DEGs, red dots represent upregulated genes, blue dots represent downregulated genes. **b** The heatmaps of top20 up-regulated and down-regulated genes, red indicates higher gene expression and green indicates lower gene expression. **c** GO enrichment bar graph of differential genes in IRI^+^ and IRI^−^ groups after transplantation in GSE151648 dataset. **d** Bar graph of KEGG enrichment of differential genes in IRI^+^ and IRI^−^ groups after transplantation in GSE151648 dataset. **e-f** GSEA enrichment analysis by GO and KEGG of differential genes in IRI^+^ and IRI^−^ group after transplantation in GSE151648 dataset

To identify the DEGs significantly related to LIRI, we downloaded the microarray expression dataset GSE151648 from the GEO database, which was the transcriptome sequencing data (RNA-seq) of Human Liver Ischemia and Reperfusion Injury, including 23 IRI^+^ patients and 17 IRI^−^ patients before and after the transplantation. The DEGs of the two groups of IRI^+^ and IRI^−^ after liver transplantation were analyzed using the online analysis tool GEO2R. A total of 161 DEGs were identified, including 114 up-regulated genes (logFC> 1 & *P*Value < 0.05) and 47 down-regulated genes in the IRI^+^ group based on |logFC| > 1, *p*Value < 0.05 (Fig. 1a-b).

### GO and KEGG enrichment analyses of DEGs

To analyze the main biological function of DEGs, functional and pathway enrichment analyses of 161 DEGs were performed using clusterProfiler [24] (Fig. 1c-d). GO analysis results showed that changes in BPs of DEGs were significantly enriched in neutrophil degranulation, neutrophil activation involved in immune response, and neutrophil mediated immunity. Changes in CCs of DEGs were mainly enriched in secretory granule membrane. Nevertheless, changes in MFs were few enriched. KEGG pathway analysis result demonstrated that the DEGs were mainly enriched in the cytokine-cytokine receptor interaction signaling pathway. To sum up, the results revealed that immune-related functions were significant in hepatic ischemia reperfusion injury.

### GSEA based on GO and KEGG analyses

It can be found that functional gene sets were not significantly different but had similar trends with genetic variation by GSEA enrichment analyses. On the basis of the GO biological process, the top 10 most significantly enriched GO terms suggested although immune-related pathways appeared few enriched in the TOP5, DEGs were significantly enriched in cytokine-cytokine receptor interaction (Fig. 1e-f), which indicated the genes related to it were highly expressed in the IRI^+^ group and might be related to inflammatory adaptability.

### LASSO regression analysis and SVM-RFE analysis

**Fig. 2:**
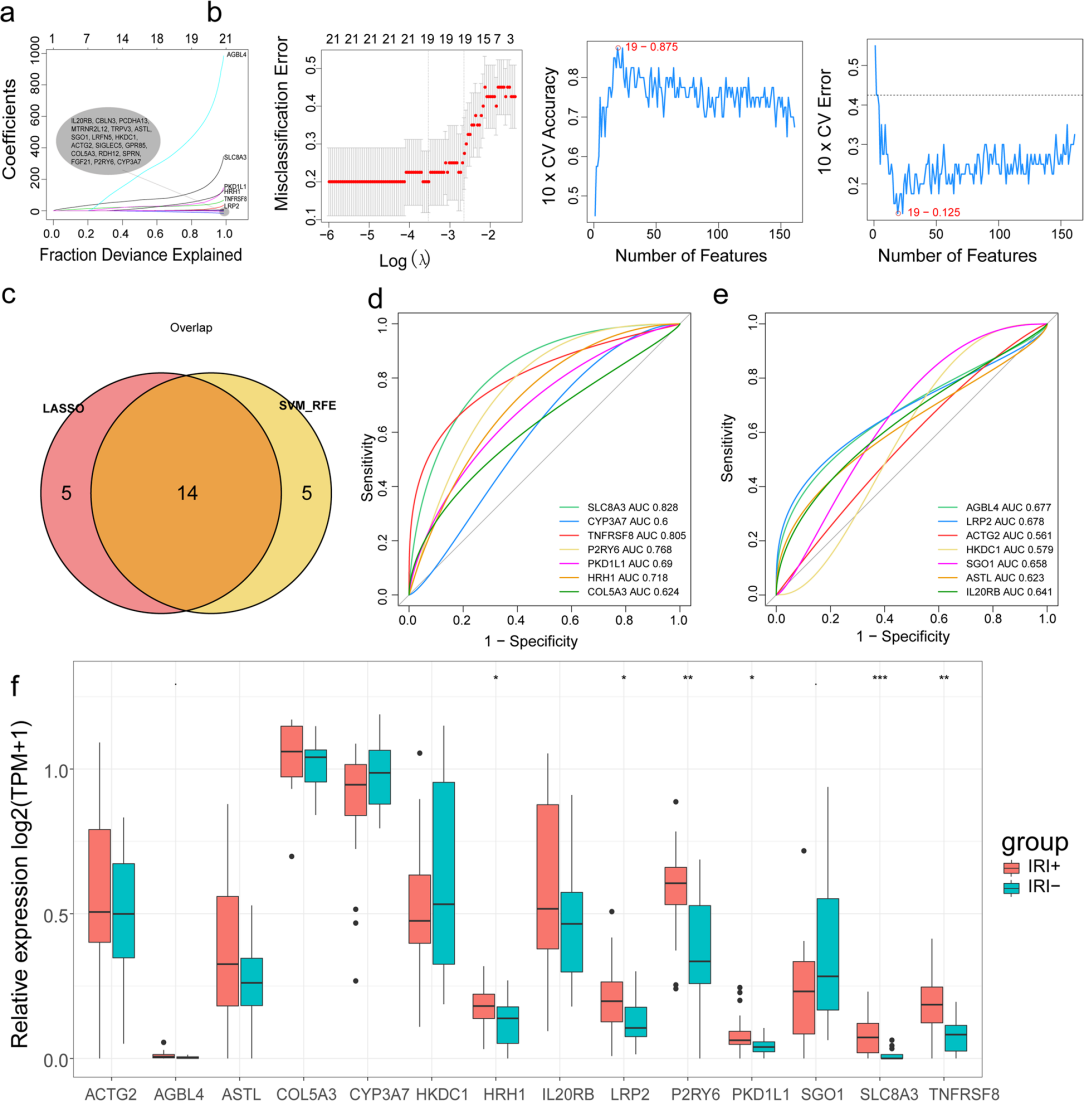
Error verification of DEGs and analyses of characteristic genes. **a** The gene coefficient diagram and cross validation error graph of LASSO regression analysis.**b** 10-fold cross-validation of LASSO, when the number of feature genes is 19,the 10 fold cross validation accuracy rate is the highest. **c** The graph of 14 intersection characteristic genes of LASSO regression analysis and SVM-RFE analysis. **d-e** ROC curves of 14 characteristic genes. **f** Expression of 13 characteristic genes between IRI+ and IRI- groups.*** indicates *p*-value < 0.001,** indicates *p*-value < 0.01, and * indicates *p*-value < 0.05

LASSO regression analysis and SVM-RFE analysis were performed on DEGs to detect the optimal combination of genes for the diagnosis of IRI+ and IRI- in the GSE151648 dataset. We performed LASSO regression analysis with 10-fold cross-validation and detected the optimal combination of 19 genes for the diagnosis of IRI+ and IRI- in the GSE151648 dataset (Fig. 2a). Meanwhile, SVM-RFE analysis with 10-fold cross-validation showed 19 combined genes for the diagnosis of IRI+ and IRI- in the GSE151648 dataset (Fig. 2b). Fourteen characteristic genes including SLC8A3, CYP3A7, TNFRSF8, P2RY6, PKD1L1, HRH1, COL5A3, AGBL4, LRP2, ACTG2, HKDC1, SGO1, ASTL, and IL20RB were selected by the intersecting the results of LASSO regression analysis and SVM-RFE analysis (Fig. 2c). Genes in the intersection sets were Subsequently, ROC curves of the 14 characteristic genes were plotted and the AUC values of SLC8A3, TNFRSF8, P2RY6, and HRH1 were greater than 0.7 (Fig. 2d-e). Furthermore,we analyzed the expressions of these 14 characteristic genes in IRI^+^ and IRI^−^ samples, and conducted a statistical test on the data of the two groups by using Wilcox.testj test to check whether there was significant difference in the expression of these 14 characteristic genes in IRI^+^ and IRI^−^ samples. Subsequently, 6 genes exhibited significantly differently expressed levels in the two groups, and the expression level of SLC8A3 was the most significant, followed by P2RY6 and TNFRSF8, and the last were HRH1, LRP2 and PKD1L1 (Fig. 2f). In summary, SLC8A3, TNFRSF8, P2RY6, and HRH1 not only with AUCs > 0.7 but also significantly differently expressed in IRI+ and IRI- groups.

### ssGSEA immuno infiltration analysis of IRI^+^ and IRI^−^ samples

**Fig. 3:**
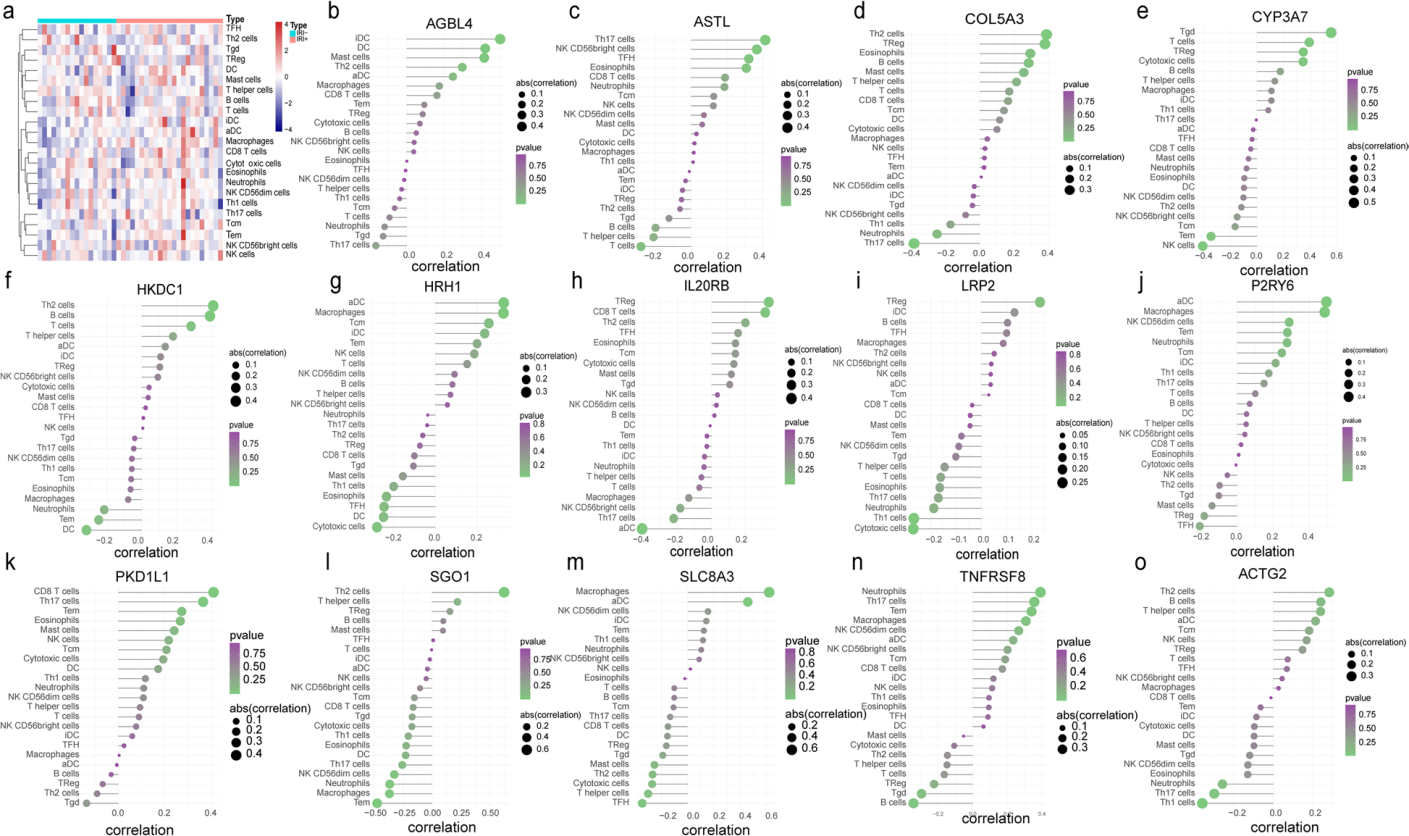
Correlation analyses of immune infiltration and hub genes with immune cells. **a** ssGSEA immune infiltration analyses of 24 IRI+ and IRI- samples. Orange is positive, blue is negative. The darker the color, the more significant the difference. **b-o** Spearman correlation analysis between 14 characteristic genes and 24 kinds of immune cells

We analyzed 24 immune-related gene sets including not only the types of immune cells, but also immune-related pathways and functions, and found that there was heterogeneity between IRI^+^ and IRI^−^ samples in part of the immune cell scores, indicating that there were differences in the level of immune cell infiltration between the two groups. The samples in IRI^−^ were rich in TFH, Tgd, NK CD56dim cells and Tem, while the samples in IRI^+^ were rich in T helper cells, T cells, neutrophils and Th1 cells (Fig. 3a).

### Correlation analysis of characteristic genes and ssGSEA scores

By Spearman correlation analysis, we found that genes such as SLC8A3, HRH1, and P2RY6 were positively correlated with macrophages. HRH1 and P2RY6 were positively correlated with aDC. PKD1L1 and TNFRSF8 were positively correlated with Th17 cells. TNFRSF8 was positively correlated with neutrophils, but PKD1L1 was negatively correlated with B cells. Thus, macrophages and neutrophils were the immune cells and worthy of attention (Fig. 3b-o).

### PPI network construction and module analysis

**Fig 4:**
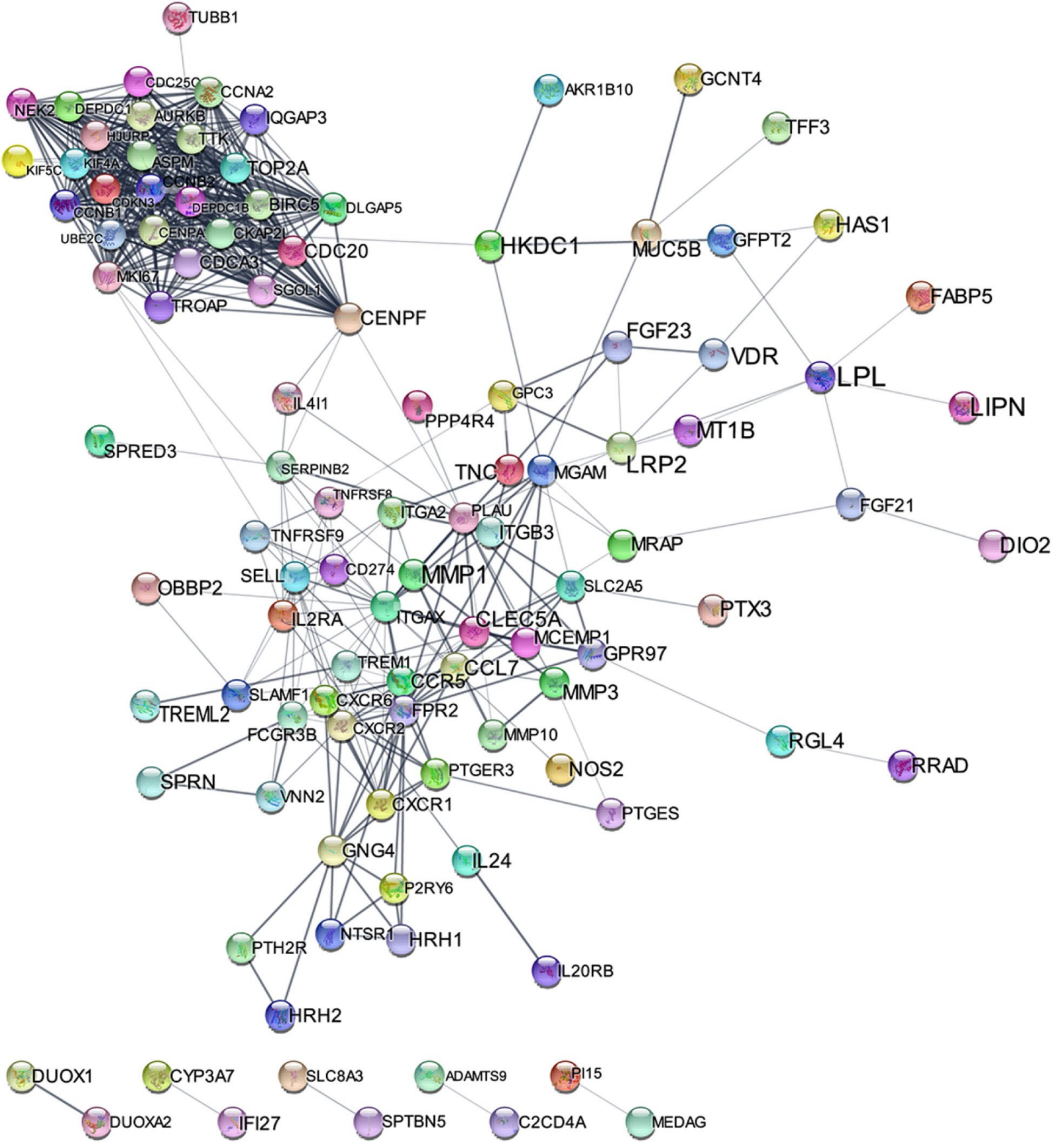
PPI network construction of DEGs. PPI network among 14 differential genes

Cytoscape was used to visualize the PPI network of 14 signature genes, MCODE was used to detect the hub modules in the PPI networks, and DAVID was used to analyze the genes involved in the hub modules (Fig. 4). Only 9 nodes were found in the protein interaction network diagram, and 5 genes (PKD1L1, COL5A3, AGBL4, ACTG2 and ASTL) were remained. In contrast, these genes were not found in the protein interaction network diagram, indicating that these genes did not interact with other proteins in the STRING database, which suggested that the genes may have not been thoroughly studied yet.

## Discussion

IRI is not only an inevitable result of organ recovery, ischemia, and reperfusion after the transplantation, but also a key factor for the organ transplantation. Therefore, it is vital to investigate the pathological and molecular mechanism of LIRI for organ transplantation and the liver injury. Based on existing research, LIRI can be divided into the ‘cold’ IRI and the ‘warm’ IRI [25]. In clinical liver transplantations, the recipient livers often suffers from warm ischemia due to self-injury, while the donor’s livers suffer from cold ischemia during ex vivo preservation. As a result, although initial cellular targets of the two IRI types might be different, they cannot be completely independent. Ischemia injury is a local process of cellular metabolic disturbance, and caused by glycogen depletion, oxygen deficiency, and adenosine triphosphate depletion. On the other hand, reperfusion injury involves both direct and indirect cytotoxic mechanisms [26]. In addition to hypoxia and metabolic disorders, immune is also related to the development and progression of LIRI [2]. The activation of liver Kupffer cells and neutrophils, the production of cytokines andchemokines, the generation of ROS, increased expression of adhesion molecules and infiltration by circulating lymphocytes and/or monocytes are immunological cascades present in both types of IRI [27]. The inflammation of the liver is critical, because the prevention of local immune activation continues to improve the IRI cascade. The main cell participating in this local immune response are KC, dendritic cells, neutrophils, T cells and NK/NKT cells [26]. Under hypoxic conditions, hepatocytes release high-mobility group box 1 (HMGB1), and it activates the inflammatory pathway via TLR4-dependent ROS production and downstream CaMK-mediated signal transduction [28]. TLR is regarded to be a promoter of tissue damage. TLR binds to damage-associated molecular patterns (DAMPs) released by damaged host cells to release an inflammatory cascade that amplifies tissue destruction [29]. Many articles have studied the relationship between LIRI and immune, but we know few about the immune cells involved in LIRI, the key factors in the LIRI process [30], and the associations between the key factors and immune cells. Efficient microarray and bioinformatics provide support to understanding the occurrence and the development of the molecular mechanisms of disease, which is necessary to explore genetic alternations and identify potential diagnostic biomarkers. Therefore, in this study, we aimed to identify the key genes, molecules and immune cells affecting LIRI to provide potential genes for subsequent biological experiments and immune cell therapeutic targets for the treatment of hepatic ischemia reperfusion injury.

In this study, we performed a comprehensive analysis of GSE151648, then we screened out 161 differentially expressed genes including 114 up-regulated genes and 47 down-regulated genes in IRI+ patients [31]. GO analyses revealed that the changes in the modules were mostly enriched in the positive regulation of neutrophil degranulation, neutrophil activation and neutrophil activation involved in immune response. KEGG enrichment analysis of DEGs revealed that LIRI involves the cytokine-cytokine receptor interaction signaling pathway. Moreover, nine hub genes of LIRI were identified with the highest scores in the protein-protein network [32]. Chen reported that agglomeration, adhesion and activation of neutrophils are important causes of hepatic ischemia reperfusion injury [33]. This is consistent with our analysis of immune cell infiltration in IRI+ and IRI- groups.

We obtained 14 characteristic genes from the intersection of the results of LASSO regression analysis and SVM-RFE analysis. The expressions of 14 characteristic genes in IRI+ and IRI- groups were analyzed, and the genes with significant differences in expression in the two groups were obtained by statistical test, including HRH1, LRP2, P2RY6, PKD1L1, SLC8A3 and TNFRSF8. According to the mark genes of 24 immune cells, we identified the heterogeneity of some immune cell infiltration in of IRI+ and IRI- groups by the ssGSEA immune cell infiltration analysis in the two groups. A spearman correlation analysis of 14 characteristic genes and 24 immune cells was performed, which indicated the genes may affect the level of immune cell infiltration. Histamine receptor 1 (HRH1) belongs to the rhodopsin-like G-protein-coupled receptor family. Its activation by histamine triggers cell proliferation, embryonic development, and tumor growth [34]. It was found that HRH1-activated macrophages polarize toward an M2-like immunosuppressive phenotype with increased expression of the immune checkpoint VISTA, rendering T cells dysfunctional to suppress immune rejection [35]. In addition to its role in the immune system, HRH1 is also a neurotransmitter in the central nervous system, which regulates the excitability of sympathetic preganglionic neurons in neonatal rats through direct postsynaptic effects by activating H1 receptors [36, 37]. LRP2 is a coreceptor used to control acoustic hedgehog signaling in development and disease [38]. Mutations in LRP2 can be used as a biomarker for personalized tumor immunotherapy [39]. P2RY6, a receptor for nucleotide uridine 5′ -diphosphate, has been implicated in various human diseases, including obesity and autoimmune diseases [40]. P2RY6 inhibits the proliferation and inflammatory response of keratinocytes induced by TPA and dramatically reduces the occurrence of tumors [41]. According to Chen’s study, SLC8A3 can protect myocardial cells against ischemia reperfusion injury in rats [42]. The relative mRNA expression of SLC8A3 was opposite to the protein levels of SLC8A3 and P-AKT. A/ R-induced H9c2 cell abnormalities were significantly improved by overexpression of activated P-AKT and SLC8A3, but were aggravated by suppressed P-AKT. In addition, SLC8A3 protein levels are positively regulated by P-AKT signaling. While, SLC8A3 silencing significantly increased apoptosis in H9c2 cells under normoxic conditions. Although the organization is different, the mechanisms could be similar. As a result, SLC8A3 may be a promising gene to reduce liver ischemia reperfusion injury.

This study constructed a PPI network to explore interactions among the selected DEGs, and only 2 hub modules and nine hub genes (SLC8A3, CYP3A7, TNFRSF8, P2RY6, HRH1, LRP2, HKDC1, SGO1, and IL20RB) were discovered by Cytoscape respectively. We can analyze the mechanism of the 9 genes from the PPI network to answer the question, how do they affect hepatic ischemia reperfusion injury. The 5 genes indicated that these genes did not interact with other proteins in the STRING database, which had not been thoroughly studied. This could also be our direction of study. However, we also need to deepen our understanding in further research. It is necessary to examine the basic expressions of these predicted DEGs and hub genes in the development of LIRI with western blot (WB), quantitative real-time PCR, immunohistochemistry, and immunofluorescence assays, etc.

## Conclusion

In our study, we obtained 14 genes related to liver ischemia reperfusion injury, including HRH1, LRP2, P2RY6, PKD1L1, SLC8A3, TNFRSF8, CYP3A7, COL5A3, AGBL4, ACTG2, HKDC1, SGO1,ASTL and IL20RB. We found neutrophil degranulation, neutrophil activation and neutrophil activation involved in immune response are enriched in immune response and revealed that LIRI involves the cytokine-cytokine receptor interaction signaling pathway. As we identified genes may affect the level of immune cell infiltration, which provides potential genes for subsequent biological experiments. These genes could be immune cell therapy targets for treatment of liver ischemia reperfusion injury.

## Data Availability

GSE151648 database (https://www.ncbi.nlm.gov/geo/query/acc.cgi?acc=GSE151648).

## Abbreviations

LIRI: Liver ischemia reperfusion injury
IRAK-4: IL1 receptor-associated kinase 4
NF-κB: Nuclear factor kappa-B
TNF-α: Tumor necrosis factor-α
TAK: Transforming growth factor-β-activated kinase
DUSP: Dual-specificity phosphatase
ALOX12: Arachidonate 12-lipoxygenase
*MAPK*: Mitogen-activated protein kinase
ATP: Adenosine triphosphate
*PI3K*: Phosphoinositide-3 kinase
mTOR: Mechanistic Target Of Rapamycin
NO: Nitric oxide
cAMP: Cyclic Adenosine monophosphate
DNA: Deoxyribo Nucleic Acid
*HDCs*: *Hepatic stellate cells*
MMPs: Matrix Metalloproteinases
NGAL: Neutrophil Gelatinase-associated Lipocalin
FMN: Mitochondrial flavin mononucleotide
GEO: Gene Expression Omnibus
DEGs: Differentially expressed genes
GO: Gene ontology
KEGG: Kyoto Encydlopedia of Genes and Genomes
ROS: Reactive oxygen species
DAMPs: Damage-associated molecular patterns
PRRs: Pattern recognition receptors
ET: Endothelin
RNA: Ribonucleic Acid
IRI: Ischemia reperfusion injury
BP: Biological process
MF: Molecular functions
CC: Cellular components
LASSO: Least absolute shrinkage and selection operator
SVM-RFE: SVM-Recursive Feature Elimination
PPI: Protein-protein interaction
